# The Efficacy of Electronic Health–Supported Home Exercise Interventions for Patients With Osteoarthritis of the Knee: Systematic Review

**DOI:** 10.2196/jmir.9465

**Published:** 2018-04-26

**Authors:** Axel Georg Meender Schäfer, Christoff Zalpour, Harry von Piekartz, Toby Maxwell Hall, Volker Paelke

**Affiliations:** ^1^ Course of Study Speech and Language Therapy, Occupational Therapy and Physiotherapy Faculty of Social Work and Health University of Applied Sciences and Arts Hildesheim Hildesheim Germany; ^2^ Institut für angewandte Physiotherapie und Osteopathie Fakultät Wirtschafts- und Sozialwissenschaften University of Applied Sciences Osnabrück Osnabrück Germany; ^3^ School of Physiotherapy and Exercise Science Faculty of Health Sciences Curtin University Perth Australia; ^4^ International Degree Programme in Media Computer Science Electrical Engineering and Computer Science University of Applied Sciences Bremen Bremen Germany

**Keywords:** osteoarthritis, knee, telemedicine, exercise, treatment outcome, review, meta-analysis

## Abstract

**Background:**

Osteoarthritis of the knee is the most common cause for disability and limited mobility in the elderly, with considerable individual suffering and high direct and indirect disease-related costs. Nonsurgical interventions such as exercise, enhanced physical activity, and self-management have shown beneficial effects for pain reduction, physical function, and quality of life (QoL), but access to these treatments may be limited. Therefore, home therapy is strongly recommended. However, adherence to these programs is low. Patients report lack of motivation, feedback, and personal interaction as the main barriers to home therapy adherence. To overcome these barriers, electronic health (eHealth) is seen as a promising opportunity. Although beneficial effects have been shown in the literature for other chronic diseases such as chronic pain, cardiovascular disease, and diabetes, a systematic literature review on the efficacy of eHealth interventions for patients with osteoarthritis of knee is missing so far.

**Objective:**

The aim of this study was to compare the efficacy of eHealth-supported home exercise interventions with no or other interventions regarding pain, physical function, and health-related QoL in patients with osteoarthritis of the knee.

**Methods:**

MEDLINE, CENTRAL, CINAHL, and PEDro were systematically searched using the keywords osteoarthritis knee, eHealth, and exercise. An inverse variance random-effects meta-analysis was carried out pooling standardized mean differences (SMDs) of individual studies. The Cochrane tool was used to assess risk of bias in individual studies, and the quality of evidence across studies was evaluated following the Grading of Recommendations, Assessment, Development, and Evaluation approach.

**Results:**

The literature search yielded a total of 648 results. After screening of titles, abstracts, and full-texts, seven randomized controlled trials were included. Pooling the data of individual studies demonstrated beneficial short-term (pain SMD=−0.31, 95% CI −0.58 to −0.04, low quality; QoL SMD=0.24, 95% CI 0.05-0.43, moderate quality) and long-term effects (pain −0.30, 95% CI −0.07 to −0.53, moderate quality; physical function 0.41, 95% CI 0.17-0.64, high quality; and QoL SMD=0.27, 95% CI 0.06-0.47, high quality).

**Conclusions:**

eHealth-supported exercise interventions resulted in less pain, improved physical function, and health-related QoL compared with no or other interventions; however, these improvements were small (SMD<0.5) and may not make a meaningful difference for individual patients. Low adherence is seen as one limiting factor of eHealth interventions. Future research should focus on participatory development of eHealth technology integrating evidence-based principles of exercise science and ways of increasing patient motivation and adherence.

## Introduction

As a consequence of demographic, epidemiological, and social changes, the need for chronic care increases while health care capacities decrease [1]. This requires a change in how care is delivered [2]. As a shift from hospital care to home care is observed, self-management plays an increasingly important role to manage or improve the health of patients [3]. At the same time, home care and home therapy need to be well coordinated and consistent with quality standards [1].

### Epidemiology and Consequences of Osteoarthritis of the Knee

Osteoarthritis of the knee (OAK) is an example of a chronic disease, where self-management and home therapy are an important part of health care. Following low back pain and neck pain, osteoarthritis (OA) in general is the third most common musculoskeletal disease worldwide [4]; global prevalence of OAK for persons older than 60 years is estimated at 33.6% for women and 24.3% for men [5]. Affected individuals and their families have to adapt to the disease, loss of mobility, and diminished quality of life (QoL), which are the main contributors to personal suffering. Pain, joint stiffness, instability, and decreased physical function are the major drivers for OA-related activity decline and disability [6]. As a consequence, patients with OA are at a higher risk of obesity, cardiovascular disease, and death compared with the general population [7].

### Exercise for Osteoarthritis of the Knee

As mechanical factors are the main drivers for the pathogenesis of OAK, a positive response to exercise interventions and increased physical activity (PA) can be expected. A recent systematic review has shown short-term clinical meaningful improvements of pain and physical function following exercise interventions [8]. However, access to facilities offering such therapies is restricted because of the patients’ mobility limitations, transport problems, and time constraints, especially in rural areas. Furthermore, the increase of chronic disease puts further strain on limited health care resources accelerating the shift toward home-based interventions and self-management.

Home exercise interventions include targeted physical activities aiming to improve muscle strength, joint range of motion, proprioception, and aerobic capacity; of these lower limb strengthening and isolated quadriceps training seem to have the largest effect on pain and physical function [8]. High intensity training results in greater beneficial effects on pain and physical function, eg, strength increase of knee extensors should be at least 30% to 40% to have a beneficial effect [9]. To achieve such a magnitude, physiologic principles of load progression need to be considered. The positive effects of increased muscle strength may be because of the positive influence on biomechanics, decreasing joint load, and focal stress on the articular cartilage [8].

Physical deconditioning and risk of obesity are closely associated with OAK [7]. Aerobic exercises may counteract these factors by leading to better overall fitness and supporting weight loss. Aerobic exercise leads to an increased peak oxygen uptake, which is inversely related to morbidity and mortality and reduces effort for submaximal daily tasks [8].

In patients with OAK, malalignment and altered kinematics may cause unequal distribution of load within the joint, which is seen as one driver for onset and progression of OAK [10]. Proprioceptive training such as stepping, standing, walking, balancing, and training of joint position sense may improve proprioceptive capacity and joint function [11].

### Electronic Health Interventions

Adherence to home exercise programs is however low [12], and it seems difficult to achieve effective training intensity without adequate support and motivation. Electronic health (eHealth) technology is seen as a promising possibility to overcome these limitations [13]. eHealth-supported, home-based interventions can prevent and rehabilitate or treat many chronic conditions by providing patient education, instructions for self-management, motivation, monitoring, feedback, and enabling communication [13]. These features may enhance patient motivation and promote adherence to home exercise interventions. One example for such an eHealth intervention is the Internet-based program “join2move” [14] that includes education, exercise instruction, goal setting, time contingent exercise increase, and positive reinforcement via electronic reminders.

Although more than 43,000 health-related apps are available in the US Apple Store alone [15], the evidence base for efficacy and efficiency of many existing eHealth-assisted interventions is not sufficient. The aim of this systematic review was therefore to investigate the efficacy of eHealth-supported home-exercise interventions in the treatment of patients with OAK.

## Methods

### Protocol and Registration

Methods of literature search and data analysis were specified in advance and documented in a protocol. The protocol was registered under CRD42017072079 (PROSPERO CRD register). This systematic review is reported in accordance with the Preferred Reporting Items for Systematic Reviews and Meta-Analyses recommendations [16].

### Eligibility Criteria

Randomized controlled trials (RCTs) and controlled clinical trials (CCTs) investigating eHealth-supported home exercise interventions compared with no treatment or other treatments for patients with symptomatic unilateral or bilateral OAK were included. Diagnosis of OAK was based on self-report, radiography, clinical signs, or physician diagnosis. All other forms of arthritis were excluded. Studies with all types of eHealth-supported exercise interventions were included. Outcomes considered in this review were pain, function, and QoL. Studies had to be published English or German. Date limitations were not used.

### Information Sources

The following databases were searched in July 2017: CENTRAL, MEDLINE via PubMed, CINAHL, PEDro, and journal websites. Additionally, reference lists of included studies were hand-searched. Date last searched was July 27, 2017.

### Search Strategy

Databases were searched with the keywords Knee Osteoarthritis, Exercise AND eHealth, and RCT OR CCT and their related Medical Subject Heading and synonyms.

The terms animal OR animals and arthroplast* were used to build an exclusion filter.

The boolean operators “OR,” “AND,” and “NOT” were used to build the search strategy. Detailed search strategies for electronic databases are presented in Multimedia Appendix 1.

### Study Selection

Title, keywords, abstracts, and full-texts were assessed to establish whether the study met the prespecified eligibility criteria relating to population, intervention, and study design. A checklist was used to assess eligibility criteria. Eligibility was assessed independently by two review authors (AS and CZ), and disagreements were resolved by consensus. For each selected study, the full text was retrieved for final assessment.

### Data Collection Process

Data were extracted for study design, participant characteristics, intervention, control, types of outcome measures, follow-up, outcomes, and funding using a standardized form. One author (AS) extracted the data; this was checked by a second author (CZ). Disagreements were resolved by discussion. For each outcome, means, SDs, 95% CI, and *P* values were collected for each point of measurement. When necessary, SDs were calculated using available data (eg, CI) or information presented in graphical format.

### Data Items

Data were retrieved for the following variables: study type; patient characteristics such as age, sex, and diagnosis; type of diagnosis (self-report, radiography, signs, and symptoms); the intervention (type of exercise intervention and eHealth technology, frequency and duration of sessions, and duration of therapy); the control intervention (type of intervention, frequency and duration of sessions, and duration of therapy); outcomes (construct, measurement instrument, length of follow-up, and points of measurement); and funding sources.

### Risk of Bias in Individual Studies

The Cochrane risk of bias assessment method was used to rate the risk of bias in individual studies [17]. Two authors (AS and HvP) independently assessed the risk of bias of the included studies, and disagreements were resolved by consensus. The following bias sources were assessed: random sequence generation, allocation concealment, blinding of participants and personnel, blinding of outcome assessment, incomplete outcome data, selective outcome reporting, and other bias (such as recruitment bias in cluster RCTs or unbalanced groups).

Review Manager 5.3.5 (The Nordic Cochrane Centre) was used to generate a risk of bias figure.

### Summary Measures

For continuous data, standard mean differences (SMDs) and 95% CIs were calculated from means and SDs using the following formula: SMD=mean difference/pooled SD.

Calculations were conducted with Review Manager 5.3.5 software (The Nordic Cochrane Centre). Not reported SDs were calculated with the calculator tool of Review Manager. SMDs of 0.2, 0.5, and 0.8 were rated as small, moderate, and large, respectively [18].

According to the guidelines for summary of findings tables [19], SMDs were translated in absolute mean differences by multiplying SMDs with a control group baseline SD extracted from one representative study and dividing it by the maximum points achievable on this measurement scale. A study was judged as representative when it represented the target population to a high degree and had a large weight within the meta-analysis. Relative differences were calculated dividing the absolute benefit by the representative control group baseline mean.

### Synthesis of Results

Data from multiple studies were pooled in a meta-analysis using a random-effects model. I^2^ statistic was used to assess statistical heterogeneity across pooled studies. Values of 25%, 50%, or 75% were considered as low, moderate, or high level of heterogeneity, respectively [20].

### Risk of Bias Across Studies

The Grading of Recommendations, Assessment, Development, and Evaluation (GRADE) approach was used to evaluate the quality of evidence across studies for each outcome using the following predefined criteria [21]:

1. Inconsistency (downgraded if I^2^≥50%)
2. Indirectness (downgraded if clinically heterogeneous)
3. Imprecision: downgraded if the pooled sample size was below the calculated sample size of an adequately powered single trial (optimal information size) [22] for each outcome. The minimal clinical important change (MCIC) is considered as delta in the power calculation. The following values are considered as MCIC for patients with OAK: pain (visual analog scale, VAS or numerical rating scale, NRS), physical function 20% improvement [23], and QoL (36-item short form survey) 12% improvement from baseline [24]. Furthermore, Guyatt et al [22] suggest downgrading for sample sizes < 400 (200 per group) or if the CI overlaps no effect but includes an important improvement.
4. Risk of bias: downgrading should be considered when a “substantial risk of bias across most of the body of evidence” is suspected [25].

Quality was rated as high, moderate, low, or very low according to the GRADE criteria [21]. The GradePro online app [26] was used to generate GRADE evidence profiles and a summary of findings tables. Quality of evidence across studies was evaluated by one author (AS) and checked by a second author (HvP). Disagreements were resolved by consensus.

### Additional Analyses

Subgroup analysis (not prespecified) was conducted for different eHealth modes of delivery (mobile apps vs telephone).

## Results

### Study Selection

**Figure 1:**
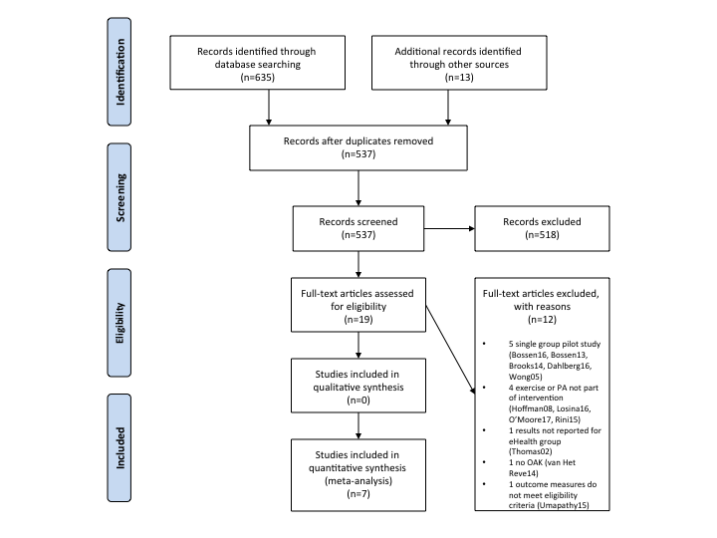
Preferred Reporting Items for Systematic Reviews and Meta-Analyses (PRISMA) flow diagram. OAK: osteoarthritis of the knee.

The literature search yielded a total of 635 records. The process of study selection is presented in Figure 1. After removing duplicates and screening of titles and abstracts, 19 full-text articles were retrieved and assessed for eligibility. Of these, 12 were excluded because of inappropriate study design, intervention, population, or outcomes.

Seven articles [14,27-32] were included, and results were pooled in a meta-analysis. One study was published twice, with outcomes pain and function reported in one article [28] and QoL in the other [29].

### Study Characteristics

The characteristics of included studies are presented in Multimedia Appendix 2. All studies were two-group RCTs. A total of 742 participants were randomized in intervention (n=376) or control (n=366) groups. Sample sizes in individual studies ranged from 34 to 211 participants. Sixty-four percent of the participants were female (473/742). Four studies included participants with unilateral or bilateral OAK [27-30,32]; one study included a mixed group with knee OA (67%), hip OA (21%), or both (12%) [14]; and one study included participants with chronic knee pain [31].

Interventions included exercises supported by mHealth (Internet-based programs or mobile apps) [14,27,31] and telephone-supported exercises [28-30,32]. Exercise interventions most commonly included strengthening exercises [28,29,31,32], walking [14,27-29], or PA reinforcement [14,27,30-32].

**Figure 2:**
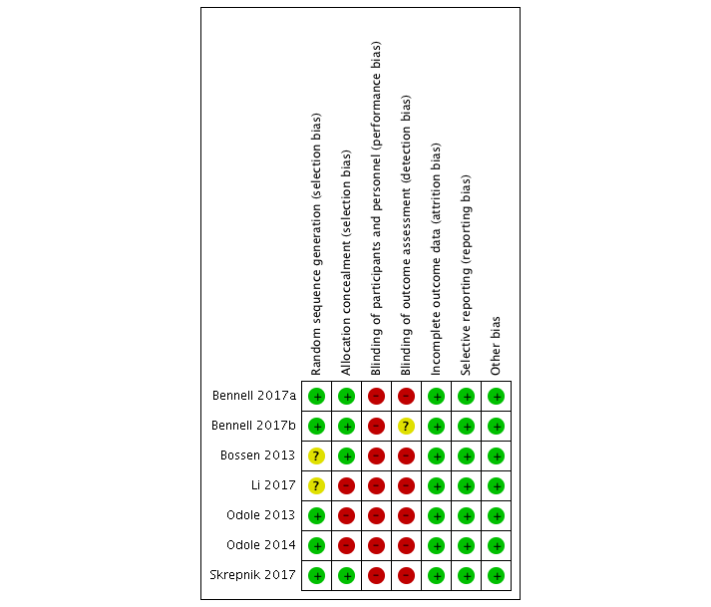
Risk of bias summary.

The eHealth component included education on topics such as exercise, healthy diet, pain management, and self-management. A counseling component, typically consisting of reminders, encouragement, and discussion of experienced barriers in varying proportions was also present. Modes of delivery included telephone calls [28-30,32], mobile apps [27], and Internet-based programs [14,31].

All studies reported pain and physical function as primary or secondary outcome measure. The most common measures of pain were the VAS or NRS used in certain studies [14,28,29,31,32], the pain subscales of the Western Ontario and MacMaster Universities Osteoarthritis Index (WOMAC) [33] used in one study [31], and the Knee injury and OA Outcome Score (KOOS) [34] used in another study [30]. Physical function was measured with the WOMAC in [14,31], the KOOS in [14,30], and the Ibadan Knee/Hip Osteoarthritis Outcome Measure in [28]. Health-related QoL was assessed with the Assessment of Quality of Life-Version 2 [35] used in [31,32], the KOOS in [14], and the WHO Quality of Life Assessment [36] was used in [29]. Other outcome measurements identified included global change, amount of PA, or steps walked.

Short-term follow-up time points of measurements included 1 month [30], 6 weeks [28,29], 3 months [14,27,31], and 6 months [32]. Long-term follow-up ranged from 9 months [32] to 12 months [14,32]. One study reported long-term outcomes at 18 months [32].

### Risk of Bias Within Studies

Risk of bias within studies was assessed using seven criteria recommended by the Cochrane Collaboration [17] (Figure 2). None of the studies reported blinding of participants, therapists, or outcome assessors. In 2 of the studies [14,30], randomization was performed, but the method of random sequence generation was not specified. Therefore, risk of selection bias was classified as unclear for these two studies. In 2 studies [28-30], allocation concealment was not reported. Attrition, reporting, or other bias was not detected in any of the included studies.

### Synthesis of Results

Pooled effect estimates including CIs are presented in this section for the outcomes pain, physical function, and health-related QoL. Calculations for absolute reduction or improvement in percentage were based on the control group baseline means (SD) from Bennell et al [32]: pain 58 (15), physical function 45 (15), and QoL 70 (10). Quality of evidence across studies was evaluated for each outcome using the GRADE approach [21]. A summary of findings table is presented in Multimedia Appendix 3.

#### Pain Short Term

**Figure 3:**
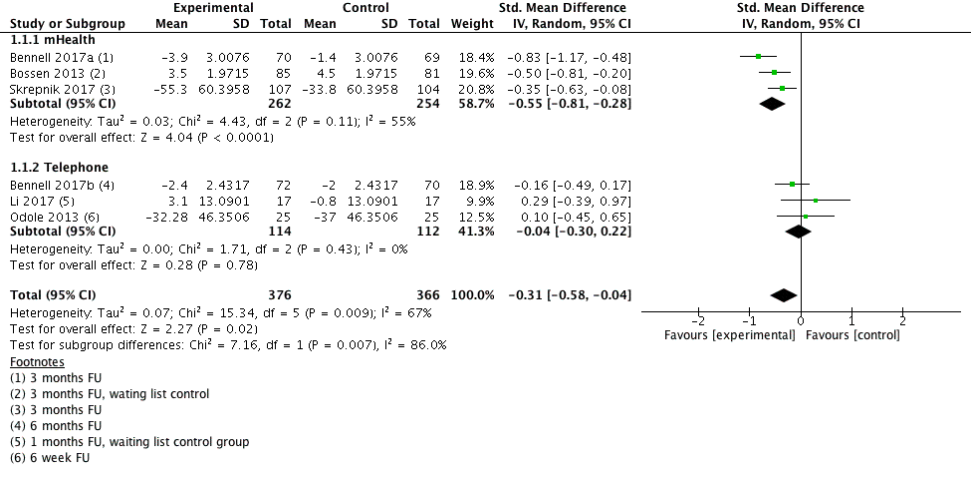
Forest plot outcome pain short-term.

All 6 studies (n=742 participants) reported data for the outcome pain intensity short term (1-6 months follow-up; Figure 3). Pooled results indicate significant benefit for eHealth-supported exercise intervention (SMD=−0.31; 95% CI −0.58 to −0.04). The effect size was small according to Cohen [18] and equals a reduction of five points (95% CI 1-9) on a 0 to 100 points pain scale (0=no pain). Heterogeneity was high with I^2^=67%. The quality of evidence for this outcome was low.

#### Pain Long Term

**Figure 4:**
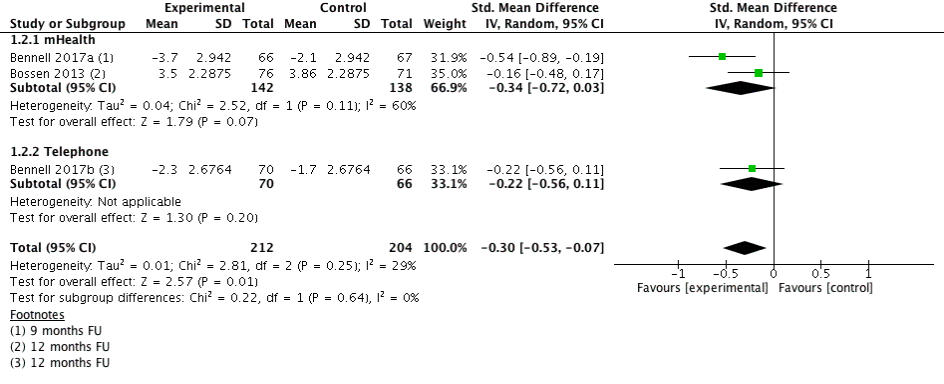
Forest plot outcome pain long-term.

Three studies (n=416 participants) provided information for the outcome pain intensity long term (9-12 months follow-up; Figure 4). Pooled effect estimates showed a significant but small beneficial effect for eHealth-supported exercise (SMD=−0.30; 95% CI −0.53 to −0.07). This translates in a reduction of five points (95% CI 1-8) on a 0 to 100 points pain scale (0=no pain). Heterogeneity was low with I^2^=29%. The quality of evidence for this outcome was moderate.

#### Physical Function Short Term

**Figure 5:**
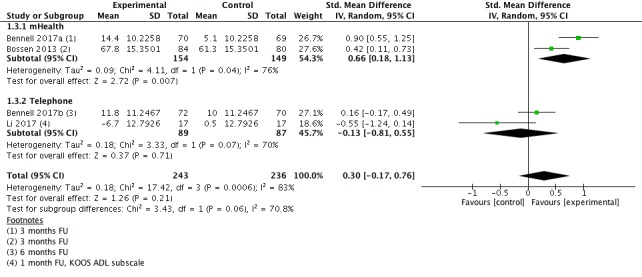
Forest plot outcome function short-term.

Four studies (n=479 participants) provided data for the outcome physical function short-term (1-6 months follow-up; Figure 5). Pooling of results from individual studies showed a nonsignificant, small beneficial effect (SMD=−0.30; 95% CI −0.76 to 0.17). This equals an improvement of four points (95% CI −3 to 11) on a 0 to 100 points physical function scale (100=full function). Heterogeneity was high with 83%. The quality of evidence for this outcome was low.

#### Physical Function Long Term

**Figure 6:**
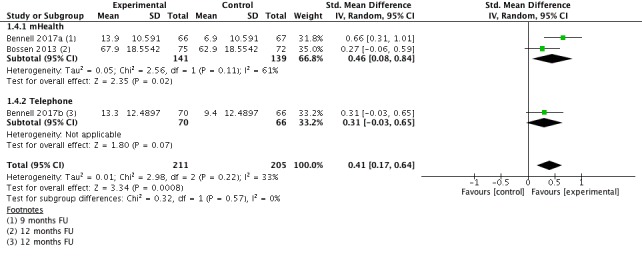
Forest plot outcome function long-term.

Data for the outcome physical function long term (9-12 months follow-up) were extracted from 3 studies (n=416 participants). Pooling the results of individual studies showed a small, significant beneficial effect favoring the intervention group (SMD=0.41; 95% CI 0.17-0.64; Figure 6). This equals an improvement of six points (95% CI 3-10) on a 0 to 100 points scale (higher scores indicate better function). Heterogeneity was moderate with I^2^=33%. The quality of evidence for this outcome was high.

#### Quality of Life Short Term

**Figure 7:**
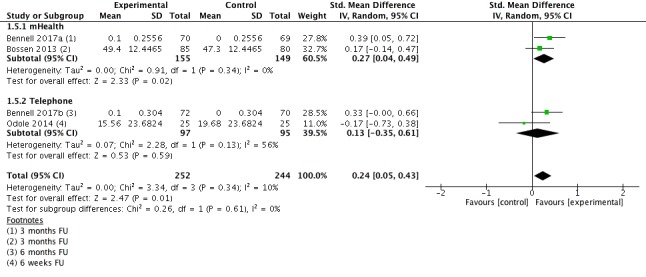
Forest plot outcome quality of life short-term.

Four studies (n=496 participants) provided information for the outcome QoL short term (3-6 months follow-up). Pooling results of individual studies showed a small, significant beneficial effect favoring the intervention (SMD=0.24; 95% CI 0.05-0.43; Figure 7). This translates in an improvement of three points (95% CI 1-4) on a 0 to 100 points scale (higher scores=better QoL). Heterogeneity was low with I^2^=10%. The quality of evidence for this outcome was moderate.

#### Quality of Life Long Term

**Figure 8:**
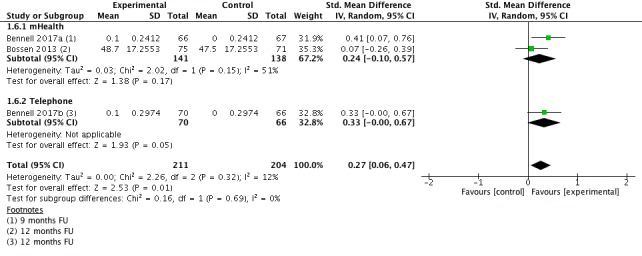
Forest plot outcome quality of life long-term.

Three studies (n=415 participants) provided data for the outcome QoL long term (9-12 months follow-up; Figure 8). Pooling results from individual studies yielded a small, significant beneficial effect favoring the intervention group (SMD=0.27; 95% CI 0.06-0.47). This corresponds to an improvement of three points (95% CI 1-4). Heterogeneity was low with I^2^=12%. The quality of evidence for this outcome was high.

#### Quality of Evidence Across Studies

For each outcome, quality of evidence was assessed using the GRADE approach [21] (Tables 1 and 2). Quality of evidence for short-term outcomes were low for pain and physical function and moderate for QoL. Reasons for downgrading one level were risk of bias because of lack of blinding of therapists, patients, and outcome assessors for all short-term outcomes. Outcomes pain and physical function were further downgraded one level because of inconsistency (I^2^ >50%). Quality of evidence for long-term outcomes was rated moderate for pain and high for physical function and QoL. The outcome pain long term was downgraded one level because of lack of blinding of therapists, patients, and outcome assessors.

#### Additional Analysis

A sensitivity analysis was conducted to assess the impact of treatment duration on heterogeneity. This was done excluding the 2 studies with the shortest intervention duration [28-30]. However, this did not substantially change the amount of observed heterogeneity between groups (pain short term: I^2^ from 67%-63%; physical function short term: 83%-79%; QoL short term: 10%-0%).

Subgroup analyses were performed to investigate whether studies with different treatment delivery modes and treatment contents differed in regards to their effect size (Table 3). Studies were classified into two groups: the first group consisted of studies where treatment was delivered via mHealth technology (mobile apps), the second consisted of studies where treatment was delivered via telephone. A significant difference was found between mHealth (SMD=−0.55) and telephone (SMD=−0.04) supported exercise studies in pooled effect estimates for the outcome pain short-term (χ^2^=7.2 *P*=.007). A substantial, but not significant difference was noted for the outcome physical function short-term between mHealth (SMD=−0.66) and telephone (SMD=0.13) supported exercise studies in pooled effect estimates for outcome pain short-term (χ^2^=3.4 *P*=.06).

**Table 1:** Grading of Recommendations, Assessment, Development, and Evaluation (GRADE) evidence profile. QoL: quality of life. RCT: randomized controlled trial.

Outcome	Quality assessment						
	Number of studies	Study design	Risk of bias	Inconsistency	Indirectness	Imprecision	Other considerations
Pain short term (follow-up: range 1 month to 6 months; assessed with self-report questionnaire 0-100 (higher numbers=more pain)	6	RCT	Serious^a^	Serious^b^	Not serious	Not serious	None
Pain long term (follow-up: range 9 months to 12 months; assessed with self-report questionnaire 0-100 (higher numbers=more pain)	3	RCT	Serious^a,^^c^	Not serious	Not serious	Not serious	None
Physical function short term (follow-up: range 1 month to 6 months; assessed with self-report questionnaire 0-100; higher numbers=better function)	4	RCT	Serious^a,^^c^	Serious^b^	Not serious	Not serious	None
Physical function long term (follow-up: range 9 months to 12 months; assessed with self-report questionnaire 0-100; higher numbers=better function)	3	RCT	Not serious	Not serious	Not serious	Not serious	None
QoL short term (follow-up: range 3 months to 6 months; assessed with self-report questionnaire 0-100; higher numbers=better QoL)	4	RCT	Serious^a^	Not serious	Not serious	Not serious	None
QoL long term (follow-up: range 9 months to 12 months; assessed with self-report questionnaire 0-100; higher numbers=better QoL)	3	RCT	Not serious	Not serious	Not serious	Not serious	None

^a^Serious risk of bias across studies because of missing blinding of therapists, patients, and outcome assessors.

^b^Heterogeneity was high with I^2^ >50%.

^c^Randomization or allocation procedure unclear for some studies.

**Table 2:** Grading of Recommendations, Assessment, Development, and Evaluation (GRADE) summary of findings. QoL: quality of life. SMD: standardized mean difference.

Outcome	Number of patients		Effect		Quality	Importance
	Electronic health–supported exercise	No or other intervention	Relative (95% CI)	Absolute (95% CI)		
Pain short term (follow-up: range 1 month to 6 months; assessed with self-report questionnaire 0-100 (higher numbers=more pain)	367	361	–^a^	SMD 0.31 SD lower (0.04 lower to 0.58 lower)	++oo Low	Important
Pain long term (follow-up: range 9 months to 12 months; assessed with self-report questionnaire 0-100 (higher numbers=more pain)	212	204	–	SMD 0.3 SD lower (0.07 lower to 0.53 lower)	+++o Moderate	Critical
Physical function short term (follow-up: range 1 months to 6 months; assessed with self-report questionnaire 0-100; higher numbers=better function)	243	236	–	SMD 0.3 SD higher (0.17 lower to 0.76 higher)	++oo Low	Important
Physical function long term (follow-up: range 9 months to 12 months; assessed with self-report questionnaire 0-100; higher numbers=better function)	211	205	–	SMD 0.41 SD higher (0.17 higher to 0.64 higher)	++++ High	Critical
QoL short term (follow-up: range 3 months to 6 months; assessed with self-report questionnaire 0-100; higher numbers=better QoL)	227	219	–	SMD 0.24 SD higher (0.05 higher to 0.43 higher)	+++o Moderate	Important
QoL long term (follow-up: range 9 months to 12 months; assessed with self-report questionnaire 0-100; higher numbers=better QoL)	211	204	–	SMD 0.27 SD higher (0.06 higher to 0.47 higher)	++++ High	Critical

^a^Indicates "not applicable".

**Table 3:** Data and analysis.

Outcome		Studies	Participants	Statistical method	Effect estimate
**1.1 Pain short term**		6	742	SMD^a^ (IV, Random, 95% CI)	−0.31 (−0.58 to −0.04)
	1.1.1 mobile health (mHealth)	3	516	SMD (IV, Random, 95% CI)	−0.55 (−0.81 to −0.28)
	1.1.2 Telephone	3	226	SMD (IV, Random, 95% CI)	−0.04 (−0.30 to 0.22)
**1.2 Pain long term**		3	416	SMD (IV, Random, 95% CI)	−0.30 (−0.53 to −0.07)
	1.2.1 mHealth	2	280	SMD (IV, Random, 95% CI)	−0.34 (−0.72 to 0.03)
	1.2.2 Telephone	1	136	SMD (IV, Random, 95% CI)	−0.22 (−0.56 to 0.11)
**1.3 Physical function short term**		4	479	SMD (IV, Random, 95% CI)	−0.30 (−0.17 to 0.76)
	1.3.1 mHealth	2	303	SMD (IV, Random, 95% CI)	0.66 (0.18 to 1.13)
	1.3.2 Telephone	2	176	SMD (IV, Random, 95% CI)	—0.13 (−0.81 to 0.55)
**1.4 Physical function long term**		3	416	SMD (IV, Random, 95% CI)	0.41 (0.17 to 0.64)
	1.4.1 mHealth	2	280	SMD (IV, Random, 95% CI)	0.46 (0.08 to 0.84)
	1.4.2 Telephone	1	136	SMD (IV, Random, 95% CI)	0.31 (−0.03 to 0.65)
**1.5 Quality of Life short term**		4	496	SMD (IV, Random, 95% CI)	0.24 (0.05 to 0.43)
	1.5.1 mHealth	2	304	SMD (IV, Random, 95% CI)	0.27 (0.04 to 0.49)
	1.5.2 Telephone	2	192	SMD (IV, Random, 95% CI)	0.13 (−0.35 to 0.61)
**1.6 Quality of Life long term**		3	415	SMD (IV, Fixed, 95% CI)	0.27 (0.06 to 0.47)
	1.6.1 mHealth	2	279	SMD (IV, Random, 95% CI)	0.24 (−0.10 to 0.57)
	1.6.2 Telephone	1	136	SMD (IV, Random, 95% CI)	0.33 (0.00 to 0.67)

## Discussion

### Principal Findings

This systematic review included six RCTs with a total of 742 participants. Pooling the results of 6 studies demonstrated that eHealth-supported exercise interventions resulted in improved pain (SMD=−0.31, 95% CI −0.58 to −0.04) and pooled results from 4 studies (n=446 participants) indicated improvement of health-related QoL (SMD=0.24, 95% CI 0.05-0.43) immediately post intervention. These treatment effects would be considered small and translate into an absolute mean improvement of 5% (95% CI 1%-9%) for pain and 3% (95% CI 1%-4%) for health-related QoL. Improvement in pain was comparable with other interventions such as nonsteroidal antiinflammatory drugs (SMD=−0.29, 95% CI −0.35 to −0.22) or strengthening (SMD=−0.32, 95% CI −0.42 to −0.23) and were superior to aquatherapy (SMD=−0.19, 95% CI −0.35 to −0.04) [37]. Fransen et al [8] demonstrated that land-based exercise resulted in higher effect sizes of −0.49 (95% CI −0.59 to −0.39) for pain and 0.52 (95% CI 0.39 - 0.64) for physical function. Results for QoL were comparable with SMD 0.28 (95% CI 0.15-0.40).

One recent meta-analysis [38] compared exercise-based telemedicine with no intervention in patients with chronic pain and found significant mean reduction in pain (mean difference=−0.57 on a 10-point scale; 95% CI −0.81 to −0.34), which corresponds to an SMD of 0.22. Improvement in physical function (SMD=−0.20, 95% CI −0.29 to −0.12) post intervention favored the intervention group. When comparing exercise-based telemedicine with usual care or exercise-based telemedicine in addition to usual care, no significant differences were observed. These effects are smaller compared with the results identified in this review. A mixed population of chronic pain patients may respond differently to eHealth-supported exercise compared with patients with OAK. One main difference is that patients with OAK have an identifiable specific structural pathology, whereas patients with chronic pain are heterogeneous in regards to pathology and contributing factors and possibly respond to a lesser extent to exercise therapy.

One important finding of this meta-analysis was that the pooled long-term outcomes from 3 studies (n=416) showed that eHealth-supported exercise resulted in reduced pain (SMD=−0.30, 95% CI −0.53 to −0.07), improved physical function (SMD=0.41, 95% CI 0.17 - 0.64), and QoL (SMD=0.26, 95% CI 0.06 - 0.47) [27-29]. These treatment effects would be considered small and translate into absolute mean improvement of 5% (95% CI 1%-8%) for pain, 6% (95% CI 6%-10%) for physical function, and 3% (95% CI 1%-4%) for QoL. These findings indicate that the effects of eHealth-supported exercise are sustainable over a 9 to 12 months period.

Although observed improvements for most long- and short-term outcomes were statistically significant in this systematic review, they may not make a relevant difference for individual patients. Minimal clinical important changes from baseline are 20% for pain (VAS or NRS) and physical function [23] and 12% for QoL [24]. These are substantially higher than absolute changes found in this meta-analysis.

Additionally, it is important to note that 2 of the 6 studies used a waiting list control group [14,30] and another 2 studies used an education control group [27,31]. It may be possible that the choice of the control group may have inflated the effect size. However, one study [30] with waiting list control group reported a nonsignificant effect for pain and function short term in favor of the control group.

One subgroup analysis per outcome comparing the effects of different treatment delivery modalities and treatment contents (mHealth and telephone) was conducted (Table 3). Studies in the subgroups differed in regards to mode of communication (automated in the mHealth subgroup vs personal in the telephone subgroup), access to the intervention (selfguided in the mHealth subgroup and fixed dates in the telephone subgroup), and contents of intervention. Although results have to be interpreted cautiously as the comparison is not based on randomization, a general trend for greater effect sizes in the mHealth group was observed that reached statistical significance for the outcome pain short-term. Possible reasons for the observed greater beneficial effect of mHealth interventions are stated below.

First, mHealth interventions were more complex and consisted of various elements such as information, educational material, training of self-management skills, and exercise compared with telephone interventions that typically included telephone coaching and exercises.

Second, different control group interventions may also account for a greater observed effect in studies investigating mHealth. In mHealth studies, control interventions included educational material only [31], waiting list [14], and injections plus information [27]. In comparison, control groups in the telephone studies consisted of supervised physiotherapy in 2 studies [28,29,32] and waiting list in one study [30].

Third, treatment duration was shorter in 2 of the telephone studies with 4 and 6 weeks [28-30] compared with 3 months in the mHealth studies [14,23,27,28,31]. Each of these factors alone or in combination could have contributed to the observed differences in effect size.

### Limitations

In this section, limitations at study and outcome level, as well as limitations of the review process, are discussed.

#### Risk of Bias at Study Level

Risk of bias was low for 4 of the included studies [14,27,31,32] and moderate for the remaining 2 studies [28-30]. Lack of blinding of patients, therapists, and outcome assessors was noted in all of the studies. Although blinding of participants and therapists to treatment modality is difficult to achieve in exercise interventions, lack of blinding may nonetheless introduce overestimation of effects and should therefore be assessed [17]. Blinding of outcome assessors and statisticians would have been possible. However, only one study reported blinding of the statistician and that patients were unaware of the study hypothesis [31]. In the 2 studies with the highest risk of bias [28-30], allocation concealment was not reported; in one study [28,29], adequate random sequence generation was unclear. As the net effect of these 2 studies on the pooled effect size is in favor of the control group, the overall risk of bias is judged as low.

#### Overall Quality of Evidence

Quality of evidence across studies was assessed high for the outcomes physical function long term and QoL long term, moderate for pain long term and QoL short term, and low for pain short term and physical function short term.

Reasons for downgrading the quality of evidence was risk of bias and imprecision. For the outcomes pain short term, pain long term, physical function short term, and QoL short term, quality of evidence was downgraded one level because the risk of bias was assessed as serious. Reasons were lack of blinding and unclear allocation concealment across studies that may have introduced some overestimation of the results.

Quality was downgraded one level because of substantial inconsistency for the short-term outcomes pain (I^2^=67%) and physical function (I^2^=83%). Some reasons for inconsistency have been described above and include differences in treatment delivery modes (mHealth vs telephone), treatment duration, and control treatments. The exercise component of the eHealth intervention also varied between studies. Strengthening and reinforcement of PA was used in 3 studies [28,29,31,32], reinforcement of aerobic exercise such as walking or cycling in 2 studies [14,27], and reinforcement of general PA in one study [30].

Further reasons for inconsistency may include heterogeneity between study populations. One study [31] included patients with chronic knee pain. The proportion of patients with arthritis was probably high in this study as the inclusion criteria included age above 50 years, knee pain during walking, and more than 20 points on the WOMAC physical function subscale. Another study [14] included patients with hip and knee OA, but the majority of patients (79%) had OAK. Additionally, diverse cultural and socioeconomic backgrounds of participants coming from Australia, the Netherlands, the United States, Canada, and Nigeria may have contributed to the heterogeneity of study participants.

#### Limitations in the Review Process

Some limitations regarding the review process should be mentioned. These include that only studies published in English or German language were considered. Studies published in other languages could not be considered and were potentially overlooked. Studies investigating the effect of eHealth interventions are rapidly increasing; four published study protocols could be identified that matched the eligibility criteria of this systematic review [39-42]. It is possible that results from these ongoing studies may change the findings of the meta-analysis in this review.

### Conclusions

Overall, eHealth-supported exercise interventions demonstrated beneficial small short- and long-term effects on pain, physical function, and QoL in patients with OAK. These effects may be too small to make a relevant difference for individual patients. The quality of evidence was low to moderate for short-term outcomes, therefore future trials are likely to change the results for short-term outcomes. The quality of evidence for long-term outcomes were moderate to high; it seems unlikely that future studies may change results substantially.

Taking into account the balance between benefits and harm, the magnitude of effects, the importance of outcomes, the quality of evidence, the values and preferences of patients, and cost-effectiveness [25], the following recommendation is put forward:

In patients with OAK, clinicians should consider using eHealth interventions to support home exercise and self-management (weak recommendation, moderate quality of evidence).

This recommendation places a high weight on the positive balance of (small) benefits against possible adverse events and on patient’s values and preferences. Less weight is placed on implementation barriers because of lack of training and financial incentives of health care providers.

## Appendix

Multimedia Appendix 1 Search strategies.

Multimedia Appendix 2 Characteristics of included studies.

Multimedia Appendix 3 Electronic health (eHealth)-supported exercise compared with no or other interventions for knee osteoarthritis GRADE summary of findings table.

## Abbreviations

eHealth: electronic health
CCT: controlled clinical trial
GRADE: Grading of Recommendations, Assessment, Development, and Evaluation
KOOS: Knee Osteoarthritis Outcome Score
MCIC: minimal clinical important change
NRS: Numerical Rating Scale
OA: osteoarthritis
OAK: osteoarthritis of the knee
QoL: quality of life
PA: physical activity
RCT: randomized controlled trial
SMD: standardized mean difference
VAS: Visual Analog Scale
WOMAC: Western Ontario and MacMaster Universities Osteoarthritis Index
