# Supplementary material for: The Efficacy of Electronic Health–Supported Home Exercise Interventions for Patients With Osteoarthritis of the Knee: Systematic Review
Source: J Med Internet Res. 2018 Apr 26;20(4):e152. doi: 10.2196/jmir.9465 (PMC5945993; doi:10.2196/jmir.9465)
Supplement: Multimedia Appendix 1 [file jmir_v20i4e152_app1.pdf]

Appendix A1 Search Strategies

Medline via PubMed: Date run 27th of July 2017

**Recent queries in pubmed Search**	**Query**	**Items found**
#5	(#1 AND #2 AND #3) NOT #4	341
#4	"Search (animals OR animal OR arthroplast*)"	6320310
#3	"Search ((exercis* OR exertion* OR training* OR sport OR ((strength* OR isometric* OR isotonic* OR isokinetic* OR aerobic* OR endurance OR weight*) AND (exercis* OR train*)) OR (muscle strength*) OR (physical endurance) OR physiotherap* OR therapy OR therapies OR rehabilitation OR (selfmanagement OR self-management)))"	9062272
#2	"Search (((((internet-based) OR (electronic (interventions OR aids)) OR (computer (interventions OR aids)) OR Internet-mediated OR Web-based OR (smart-phone application) OR (mobile application) OR (app OR apps) OR (computer assisted technology) OR (ehealth OR e-health OR mhealth OR telehealth OR telemedicine OR telerehabilitation) OR (personal digital assistant*) OR ((online OR web) and (education* OR train*)) OR webinar OR (tablet* computer*) OR (ipad* OR iphone* OR ipod*) OR (ios OR android) OR ((mobile* OR cell OR smart) and phone*))OR (social media*) OR (twitter* OR tweet*) OR email* OR facebook* OR (multimedia messag*) OR (text messag*) OR ((sms OR mms) AND messag*))))"	259503
#1	"Search (knee osteoarthritis [MeSH] OR ((arthrosis OR arthritis OR osteoarthr* OR OA OR degeneration OR degenerative OR arthralgia) AND knee) OR ((arthrose knie) OR gonarthrose OR gonarthrosis))"	57276

CENTRAL search strategy: Date run 27th of July 2017

ID	Search	Hits
#1	MeSH descriptor: [Osteoarthritis, Knee] explode all trees	2332
#2	((arthrosis OR arthritis OR osteoarthr* OR OA OR degeneration OR degenerative OR arthralgia) and knee) OR ((arthrose knie) OR gonarthrose OR gonarthrosis)	6939
#3	#1 OR #2	6939
#4	MeSH descriptor: [Telemedicine] explode all trees	1901
#5	(internet-based) OR (electronic (interventions OR aids)) OR (computer (interventions OR aids)) OR Internet-mediated OR Web-based OR (smart-phone application) OR (mobile application) OR (app OR apps) OR (computer assisted technology) OR (ehealth OR e-health OR mhealth OR telehealth OR telemedicine) OR (personal digital assistant*) OR ((online OR web) and (education* OR train*)) OR webinar OR (tablet* computer*) OR (ipad* OR iphone* OR ipod*) OR (ios OR android) OR ((mobile* OR cell OR smart) and phone*) OR (social media*) OR (twitter* OR tweet*) OR email* OR facebook* OR (multimedia messag*) OR (text messag*) OR ((sms OR mms) and messag*)	33376
#6	#4 OR #5	33564
#7	MeSH descriptor: [Exercise] explode all trees	19057
#8	(exercis* OR exertion* OR training* OR sport OR ((strength* OR isometric* OR isotonic* OR isokinetic* OR aerobic* OR endurance OR weight*) and (exercis* OR train*)) OR (muscle strength*) OR (physical endurance) OR physiotherap* OR therapy OR therapies OR rehabilitation OR (selfmanagement OR self-management))	575691
#9	#7 OR #8	576272
#10	#6 and #9	22312
#11	#3 and #10	460
#12	animals OR animal OR arthroplast*	35423
#13	#11 not #12	161

CINAHL Database Search strategy: Date run 27th of July 2017

ID	Search	Hits
S1	(MH "Osteoarthritis, Knee") OR (((MH "Osteoarthritis") OR arthrosis OR arthritis OR osteoarthr* OR OA OR degeneration OR degenerative OR arthralgia) AND ((MH "Knee Joint") OR (MH "Knee") OR knee)) OR ((arthrose knie) OR gonarthrose OR gonarthrosis))	8,539
S2	(MH "Telehealth")	3,657
S3	(internet-based) OR (electronic (interventions OR aids)) OR (computer (interventions OR aids)) OR Internet-mediated OR Web-based OR (smart-phone application) OR (mobile application) OR (app OR apps) OR (computer assisted technology) OR (ehealth OR e-health OR mhealth OR telehealth OR telemedicine OR telerehabilitation) OR (personal digital assistant*) OR ((online OR web) and (education* OR train*)) OR webinar OR (tablet* computer*) OR (ipad* OR iphone* OR ipod*) OR (ios OR android) OR ((mobile* OR cell OR smart) and phone*) OR (social media*) OR (twitter* OR tweet*) OR email* OR facebook* OR (multimedia messag*) OR (text messag*) OR ((sms OR mms) and messag*)	55,478
S4	S2 OR S3	55,478
S5	(MH "Exercise") OR (MH "Resistance Training") OR (MH "Therapeutic Exercise") OR (MH "Open Kinetic Chain Exercises") OR (MH "Rehabilitation Exercise (Saba CCC)") OR (MH "Exercise Therapy: Joint Mobility (Iowa NIC)") OR (MH "Exercise Therapy: Balance (Iowa NIC)") OR (MH "Exercise Therapy: Ambulation (Iowa NIC)") OR (MH "Exercise Therapy: Muscle Control (Iowa NIC)") OR (MH "Aerobic Exercises") OR (MH "Lower Extremity Exercises") OR (MH "Isometric Exercises") OR (MH "Isokinetic Exercises") OR (MH "Anaerobic Exercises") OR (MH "Closed Kinetic Chain Exercises")	46,142
S6	(exercis* OR exertion* OR training* OR sport OR ((strength* OR isometric* OR isotonic* OR isokinetic* OR aerobic* OR endurance OR weight*) AND (exercis* OR train*)) OR (muscle strenth*) OR (physical endurance) OR physiotherap* OR therapy OR therapies OR rehabilitation OR (selfmanagement OR self-management))	968,121
S7	S5 OR S6	968,121
S8	S4 AND S7	15,086
S9	S1 AND S8	30

PEDro Advanced Search: Date run 27th of July 2017

1. Title and Abstract: osteoarthritis knee exercise
2. Match all search terms (AND)
3. Method: Clinical trial

27th July 2017: 366 records

Journal Websites: Date run 27th of July 2017

1. Journal of Telemedicine and Telecare (http://journals.sagepub.com/loi/jtt?expanded=2000-2009&expanded=2005): Osteoarthritis knee – 13 records
2. JMIR Publications (http://jmirpublications.com). Keywords: (Osteoarthritis knee) AND exercise: 95 records.
