# Supplementary material for: The Efficacy of Electronic Health–Supported Home Exercise Interventions for Patients With Osteoarthritis of the Knee: Systematic Review
Source: J Med Internet Res. 2018 Apr 26;20(4):e152. doi: 10.2196/jmir.9465 (PMC5945993; doi:10.2196/jmir.9465)
Supplement: Multimedia Appendix 2 [file jmir_v20i4e152_app2.pdf]

ID	Country	Design	Population	Intervention	Control	Follow-up	Outcomes	Funding
[31] Bennell 2017	Australia	Randomized controlled trial (RCT)	148 participants with chronic knee pain (self-report), mean age 61 years, 56% female	n=74<br><br>Internet-delivered educational material, exercise and pain coping skills training (PainCOACH).<br><br>Exercise: home lower limb strengthening, reinforcement of physical activity levels<br><br>Duration: 3 months	n=74<br><br>Internet-based educational material	3 and 9 months	Primary: pain during walking (NRS[a]), physical function (WOMAC[b]).<br><br>Secondary: knee pain (WOMAC), quality of life (Assessment of Quality of life instrument), global change (overall, pain, and functional status; 7-point Likert scale)	National Health and Medical Research Council (NHMRC)
[14] Bossen 2013	Nether-lands	RCT	199 participants with knee OA[c], hip OA or both (self-reported), 79% knee OA, mean age 62 years, 64% female	n=100<br><br>Web-based behavior graded activity program (Join2move)<br><br>Exercise: reinforcement of self-selected activities cycling or walking, home	n=99<br><br>Waiting list	3 and 12 months	Primary: physical activity (Physical Activity Scale for the Elderly, PASE), physical function (KOOS[d] or Hip disability Osteoarthritis Score, HOOS), self-perceived effect (7-point	Bossen and Veenhof created Join2-move

ID	Country	Design	Population	Intervention	Control	Follow-up	Outcomes	Funding
				strengthening and stretching exercises<br><br>Duration: 3 months			Likert scale).<br><br>Secondary: pain (NRS), fatigue (NRS), quality of life (QoL; KOOS or HOOS)	
[27]<br><br>Skrepnik 2017	United States	RCT	211 participants with unilateral knee OA (diagnostic criteria not reported), mean age 63 years, 50% female	n=107<br><br>Hyaloranon injections. Information on the benefits of walking, wearable activity monitor, activity enhancing app (OA Go).<br><br>Exercise: walking<br><br>Duration: 3 months	n=104<br><br>Hyaloranon injections. Information on the benefits of walking	3 months	Primary: mobility (Steps per day).<br><br>Secondary: distance walked (6-min walk test), pain (6-min walk test), patient activation (patient activation measure-13)	Sanofi Bio-surgery
[32]<br><br>Bennell 2017	Australia	RCT	168 participants with knee OA (clinical criteria), mean age 62 years, 63%	n=84<br><br>Physical therapy (PT) visits, home exercise, and telephone coaching<br><br>Exercise:	n=84<br><br>PT visits, home exercise	6, 12, and 18 months	Primary: pain (VAS[e]), physical function (WOMAC).<br><br>Secondary: physical Activity	NHMR, various financial support

ID	Country	Design	Population	Intervention	Control	Follow-up	Outcomes	Funding
			female	home lower limb strengthening, reinforcement of physical activity<br><br>Duration: 6 months			(PASE), QoL (assessment of QoL), 7-point global rating of change (overall, pain, physical function)	
[30]<br><br>Li 2017	Canada	Randomized, delayed control design	34 participants with knee OA (physician confirmed), mean age 56 years, 82% female	n=17<br><br>Standardized group education. Physical activity tracker. Weekly activity counseling via telephone.<br><br>Exercise: reinforcement of physical activity<br><br>Duration: 4 weeks	n=17<br><br>Delayed group receiving the same program 1 month later	1 and 2 months	Primary: mean moderate to vigorous physical activity per day.<br><br>Secondary: pain (KOOS), function (KOOS)	n. r.
[28, 29]<br><br>Odole 2013, 2014	Nigeria	RCT	50 participants with knee OA (diagnostic criteria not reported), mean age 56 years, 48% female	n=25<br><br>Exercise program with telephone coaching<br><br>Exercise: stretching, strengthening, walking	n=25<br><br>Same exercise program in clinic	2, 4, and 6 weeks	Primary: pain (VAS), function (Ibadan Knee/Hip Osteoarthritis Outcome Measure), QoL (World Health Organization	n. r.

ID	Country	Design	Population	Intervention	Control	Follow -up	Outcomes	Funding
				Duration: 6 weeks			Quality of Life-BREF)	

[a]Numerical Rating Scale.

[b]WOMAC: Western Ontario and MacMaster Universities Osteoarthritis Index.

[c]OA: osteoarthritis.

[d]KOOS: Knee Osteoarthritis Outcome Score.

[e]VAS: Visual Analog Scale.

14. Bossen D, Veenhof C, Van Beek KE, Spreeuwenberg PM, Dekker J, De Bakker DH. Effectiveness of a web-based physical activity intervention in patients with knee and/or hip osteoarthritis: randomized controlled trial. Journal of medical Internet research; 2013 Nov 22;15(11):e257. PMID: 24269911. doi: 10.2196/jmir.2662.
27. Skrepnik N, Spitzer A, Altman R, Hoekstra J, Stewart J, Toselli R. Assessing the Impact of a Novel Smartphone Application Compared With Standard Follow-Up on Mobility of Patients With Knee Osteoarthritis Following Treatment With Hylan G-F 20: A Randomized Controlled Trial. JMIR Mhealth Uhealth; 2017 May 09;5(5):e64. PMID: 28487266. doi: 10.2196/mhealth.7179.
28. Odole AC, Ojo OD. A Telephone-based Physiotherapy Intervention for Patients with Osteoarthritis of the Knee. International journal of telerehabilitation; 2013 Fall;5(2):11-20. PMID: 25945214. doi: 10.5195/ijt.2013.6125.
29. Odole AC, Ojo OD. Is telephysiotherapy an option for improved quality of life in patients with osteoarthritis of the knee? International Journal of Telemedicine and Applications; 2014;2014. PMID: CN-00988552. doi: 10.1155/2014/903816.
30. Li LC, Sayre EC, Xie H, Clayton C, Feehan LM. A Community-Based Physical Activity Counselling Program for People With Knee Osteoarthritis: Feasibility and Preliminary Efficacy of the Track-OA Study. JMIR Mhealth Uhealth; 2017 Jun 26;5(6):e86. PMID: 28652228. doi: 10.2196/mhealth.7863.
31. Bennell KL, Nelligan R, Dobson F, Rini C, Keefe F, Kasza J, French S, Bryant C, Dalwood A, Abbott JH, Hinman RS. Effectiveness of an Internet-Delivered Exercise and Pain-Coping Skills Training Intervention for Persons With Chronic Knee Pain: A Randomized Trial. Ann Intern Med; 2017 Apr 04;166(7):453-62. PMID: 28241215. doi: 10.7326/m16-1714.
32. Bennell KL, Campbell PK, Egerton T, Metcalf B, Kasza J, Forbes A, Bills C, Gale J, Harris A, Kolt GS, Bunker SJ, et al. Telephone Coaching to Enhance a Home-Based Physical Activity Program for Knee Osteoarthritis: A Randomized Clinical Trial. Arthritis Care Res (Hoboken); 2017 Jan;69(1):84-94. PMID: 27111441. doi: 10.1002/acr.22915.
