# Supplementary material for: The Efficacy of Electronic Health–Supported Home Exercise Interventions for Patients With Osteoarthritis of the Knee: Systematic Review
Source: J Med Internet Res. 2018 Apr 26;20(4):e152. doi: 10.2196/jmir.9465 (PMC5945993; doi:10.2196/jmir.9465)
Supplement: Multimedia Appendix 3 [file jmir_v20i4e152_app3.pdf]

Outcome<br>Number of participants (studies)	Anticipated absolute effects (95% CI)			Quality[g]	What happens[b]
	Without eHealth[a]-supported exercise	With eHealth-supported exercise	Difference		
Pain short term assessed with: self-report questionnaire 0-100 (higher numbers=more pain)<br>Number of participants: 728 (six randomized controlled trials, RCTs)	Mean pain in control groups was 58 on a 0-100 scale	Mean pain in intervention groups was 0.31 SDs lower (0.04-0.58 lower). This translates to an absolute mean reduction of five (1-9) points compared with control group on a 0-100 scale	SMD[c] 0.31 SD lower (0.4 lower to 0.58 lower)	⊕⊕◯◯<br>Low[d,e]	Absolute reduction in pain 5% (1%-9%); relative change 9% (2%-16%)
Pain long term assessed with self-report questionnaire 0-100 (higher numbers=more pain)<br>Number of participants: 416 (three RCTs)	Mean pain in control groups was 58 on a 0-100 scale	Mean pain in intervention groups was 0.30 SDs lower (0.07-0.53 lower). This translates to an absolute mean reduction of five (1-8) points compared with control group on a 0-100 scale	SMD 0.30 SD lower (0.07 lower to 0.53 lower)	⊕⊕⊕◯<br>Moderate[d,f]	Absolute reduction in pain 5% (1%-8%); relative change 9% (2%-14%)
Physical function short term<br>assessed with self-report questionnaire 0-100 (higher numbers=better function)<br>Number of participants: 479 (four RCTs)	Mean physical function in control groups was 45 on a 0-100 scale	Mean physical function in intervention groups was 0.30 SDs higher (0.17 lower to 0.76 higher). This translates to an absolute mean increase of four (−3-11) points compared with	SMD 0.3 SD higher (0.17 lower to 0.76 higher)	⊕⊕◯◯<br>Low[d,e,f]	Absolute improvement in physical function 4% (−3%-11%); relative change 9% (−7%-24%)

		control group on a 0-100 scale			
Physical function long term assessed with self-report questionnaire 0-100 (higher numbers=better function) Number of participants: 416 (three RCTs)	Mean physical function in control groups was 45 on a 0-100 scale	Mean physical function in intervention groups was 0.41 SDs higher (0.17-0.64 higher). This translates to an absolute mean increase of six (3-10) points compared with control group on a 0-100 scale	SMD 0.41 SD higher (0.17 higher to 0.64 higher)	⊕⊕⊕⊕ High	Absolute improvement in physical function 6% (3%-10%); relative change 13% (7%-22%)
Quality of Life (QoL) short term assessed with self-report questionnaire 0-100 (higher numbers=better QoL) Number of participants: 446 (four RCTs)	Mean QoL in control groups was 70 on a 0-100 scale	Mean QoL in intervention groups was 0.24 SDs higher (0.05-0.43 higher) This translates to an absolute mean increase of three (1-4) points compared with control group on a 0-100 scale	SMD 0.24 SD higher (0.05 higher to 0.43 higher)	⊕⊕⊕◯ Moderate[d]	Absolute improvement in QoL 3% (1%-4%); relative change 4% (1%-6%)
QoL long term assessed with self-report questionnaire 0-100 (higher numbers=better QoL) Number of participants: 415 (three RCTs)	Mean QoL in control groups was 70 on a 0-100 scale	Mean QoL in intervention groups was 0.27 SDs higher (0.06-0.47 higher). This translates to an absolute mean increase of three (1-5) points compared with control group on a 0-100 scale	SMD 0.27 SD higher (0.06 higher to 0.47 higher)	⊕⊕⊕⊕ High	Absolute improvement in QoL 3% (1%-4%); relative change 4% (1%-6%)

[a]eHealth: electronic health.

[b]Calculations based on the control group baseline means (SD) from Bennell, Campbell et al (2017): pain 58 (15), physical function 45 (15), and quality of life 70 (10).

[c]SMD: standardized mean difference.

[d]Serious risk of bias across studies because of missing blinding of therapists, patients, and outcome assessors.

[e]Heterogeneity was high with I2 > 50%.

[f]Randomization or allocation procedure unclear for some studies.

[g]GRADE working group grades of evidence: (1) high quality: the authors are very confident that the true effect lies close to that of the estimate of the effect; (2) moderate quality: the authors are moderately confident in the effect estimate: the true effect is likely to be close to the estimate of the effect, but there is a possibility that it is substantially different; (3) low quality: the authors confidence in the effect estimate is limited: the true effect may be substantially different from the estimate of the effect; and (4) very low quality: the authors have very little confidence in the effect estimate: the true effect is likely to be substantially different from the estimate of effect.
